# Reliability and reproducibility checklist for molecular dynamics simulations

**DOI:** 10.1038/s42003-023-04653-0

**Published:** 2023-03-14

## Abstract

We present a checklist to improve the reliability and reproducibility of molecular dynamics simulations and related methods.

Molecular dynamics (MD) simulations and related methods involving molecular docking, enhanced sampling, coarse-graining, and quantum mechanical calculations are widely used to provide mechanistic insight into biological, chemical, and physical phenomena at the atomistic or molecular level. The insights are valuable provided that appropriate convergence and reliability checks are done when analyzing the simulations. To maximize the value to the research community, sufficient information is required to allow reproduction or extension of the simulations for other applications.

Here, we present a checklist for reporting and assessing simulation data and data reproducibility (Table 1). It is our hope that this checklist, although far from extensive and subject to potential refinement in future, will serve as a clear guideline for publishing high quality computational work in *Communications Biology*. The guidelines in each section of the checklist include:

**Table 1:** Reliability and reproducibility checklist for molecular dynamics simulations.

1. Convergence of simulations and analysis
1a. Is an evaluation presented in the text to show that the property being measured has equilibrated in the simulations (e.g., time-course analysis)?
1b. Then, is it described in the text how simulations are split into equilibration and production runs and how much data were analyzed from production runs?
1c. Are there at least 3 simulations per simulation condition with statistical analysis?
1d. Is evidence provided in the text that the simulation results presented are independent of initial configuration?
2. Connection to experiments
2a. Are calculations provided that can connect to experiments (e.g., loss or gain in function from mutagenesis, binding assays, NMR chemical shifts, J-couplings, SAXS curves, interaction distances or FRET distances, structure factors, diffusion coefficients, bulk modulus and other mechanical properties, etc.)?
3. Method choice
3a. Do simulations contain membranes, membrane proteins, intrinsically disordered proteins, glycans, nucleic acids, polymers, or cryptic ligand binding?
3b. Is it described in the text whether the accuracy of the chosen model(s) is sufficient to address the question(s) under investigation (e.g., all-atom vs. coarse-grained models, fixed charge vs. polarizable force fields, implicit vs. explicit solvent or membrane, specific force field and water model, etc.?
3c. Is the timescale of the event(s) under investigation beyond the brute-force MD simulation timescale in this study that enhanced sampling methods are needed?
If **YES**, are the parameters and convergence criteria for the enhanced sampling method clearly stated?
If **NO**, is the evidence provided in the text?
4. Code and reproducibility
4a. Is a table provided describing the system setup that includes simulation box dimensions, total number of atoms, number of water molecules, salt concentration, lipid composition (number of molecules and type)?
4b. Are other parameters for the system setup described in the text, such as protonation state, type of structural restraints if applied, nonbonded cutoff, thermostat and barostat, etc.?
4c. Is it described in the text what simulation and analysis software and which versions are used?
4d. Are initial coordinate and simulation input files and a coordinate file of the final output provided as supplementary files or in a public repository?
4e. Is there custom code or custom force field parameters?
If **YES**, are they provided as supplementary files or in a public repository?

“It is our hope that this checklist, although far from extensive and subject to potential refinement in future, will serve as a clear guideline for publishing high quality computational work in *Communications Biology*.”

## Convergence of simulations and analysis

Without convergence analysis, simulation results are compromised. While it may not be possible to prove “absolute convergence”, multiple independent simulations starting from different configurations and time-course analyses can detect the lack of convergence. At least three independent simulations with statistical analysis should be performed to show that the properties being measured have converged. When presenting representative snapshots of a simulation, the corresponding quantitative analysis also needs to be presented to show that the snapshots are indeed representative.

## Connection to experiments

*Communications Biology* welcomes high-quality computational work that generates new biological insights and testable hypotheses. New experimental validation is highly encouraged but not required for publication. When new experimental validation is not provided, the physiological relevance of MD simulation results should be discussed in connection with published experimental data. It’s important to note that these criteria are in line with our current expectations for computational work but may change as the journal matures.

## Method choice

Method choice in MD simulations comprises two factors: model accuracy and sampling technique. With rapid growing computing capacity and algorithmic advances, we are now witnessing MD studies of increasingly large and complex biomolecular systems, such as those involving membrane proteins, intrinsically disordered proteins, glycans, and nucleic acids, at longer timescales. A simplified model that has been sampled well is more valuable than a large, complex model with poor convergence and statistics (see “Convergence of simulations and analysis”). As the best choice always depends on the system of interest, the authors need to justify that the chosen model, resolution, and force field are accurate enough to answer the specific question.

With respect to sampling methods, the functional relevant states of biomolecules are often separated by rugged free energy landscapes. Convergence analysis of the unbiased trajectories mentioned above may not detect slow transitions between kinetically trapped metastable states. Therefore, if the timescale of the event of interest is beyond unbiased sampling, the choice of enhanced sampling method(s) and the convergence of the enhanced sampling need to be provided.

## Code and reproducibility

At minimum, details on simulation parameters need to be provided in the Methods section, as well as simulation input files and final coordinate files. These can be provided in the Supplementary files or deposited in a suitable public repository, and should be sufficiently detailed to enable others to reproduce or extend the simulations.

Custom code and parameters that are central to the manuscript must also be made available for review and publicly accessible upon publication in compliance with editorial policies and reporting standards in the Nature Portfolio.

For manuscripts containing MD simulations or related methods, *Communications Biology* will require authors to submit their responses to the checklist for evaluation by the editors and reviewers, and to update the checklist when going through revisions.

We hope that the guidelines and checklist presented here will be helpful to authors, referees and, ultimately, readers of work involving molecular simulations. We welcome feedback—please get in touch by emailing commsbio@nature.com.

We are grateful to our Editorial Board Member Yun Lyna Luo, Western University of Health Sciences, Pomona, California, for her assistance in developing these guidelines and contributing to the writing of this Editorial.

